# Lindgomycin, an Unusual Antibiotic Polyketide from a Marine Fungus of the Lindgomycetaceae

**DOI:** 10.3390/md13084617

**Published:** 2015-07-27

**Authors:** Bin Wu, Jutta Wiese, Antje Labes, Annemarie Kramer, Rolf Schmaljohann, Johannes F. Imhoff

**Affiliations:** 1 GEOMAR Helmholtz Centre for Ocean Research Kiel, 24105 Kiel, Germany; E-Mails: wubin@zju.edu.cn (B.W.); jwiese@geomar.de (J.W.); alabes@geomar.de (A.L.); akramer@geomar.de (A.K.); rschmaljohann@geomar.de (R.S.); 2 Ocean College, Zhejiang University, Hangzhou 310058, China

**Keywords:** marine fungi, lindgomycin, antibiotic, MRSA, marine natural products

## Abstract

An unusual polyketide with a new carbon skeleton, lindgomycin (1), and the recently described ascosetin (2) were extracted from mycelia and culture broth of different Lindgomycetaceae strains, which were isolated from a sponge of the Kiel Fjord in the Baltic Sea (Germany) and from the Antarctic. Their structures were established by spectroscopic means. In the new polyketide, two distinct domains, a bicyclic hydrocarbon and a tetramic acid, are connected by a bridging carbonyl. The tetramic acid substructure of compound **1** was proved to possess a unique 5-benzylpyrrolidine-2,4-dione unit. The combination of 5-benzylpyrrolidine-2,4-dione of compound **1** in its tetramic acid half and 3-methylbut-3-enoic acid pendant in its decalin half allow the assignment of a new carbon skeleton. The new compound **1** and ascosetin showed antibiotic activities with IC_50_ value of 5.1 (±0.2) µM and 3.2 (±0.4) μM, respectively, against methicillin-resistant *Staphylococcus aureus.*

## 1. Introduction

Marine-derived fungi living in a stressful habitat are of great interest as new, promising sources of biologically active products. Since marine organisms live in a biologically competitive environment with unique conditions of pH, temperature, pressure, oxygen, light, nutrients, and salinity, the chemical diversity of the secondary metabolites from marine fungi is considerably high [1,2,3,4,5,6,7]. Various new genera were described from marine resources, however, marine isolates of known taxa reveal to be potent producers of novel chemistry as well. The fungal family Lindgomycetaceae (Pleosporales, Dothideomycetes) was introduced by Hirayama *et al*. [8] to accommodate four new species of the genus *Lindgomyces*. Another three *Lindgomyces* species were added by Raja *et al*. [9,10], and these authors also included *Massariosphaeria typhicola*, a closely related species, into the Lindgomycetaceae. Later on, the newly isolated species *Lolia aquatica* was added [11] and *Clohesiomyces aquaticus* was shown to belong to this family according to sequence data [12]. All members of the Lindgomycetaceae were isolated from submerged parts of decaying wood and plant material in a freshwater environment. Little is known on their metabolic capabilities and on their production of bioactive compounds. The only available data are from Raja *et al*. [10], who found a fatty acid, 6*E*,9*E*-octodecadienoic acid, and ergosterol peroxide as the major chemical compounds in *Lindgomyces angustiascus*.

The naturally occurring pyrrolidine-2,4-dione (tetramic acid) derivatives originating from a variety of marine and terrestrial species have attracted a great deal of interest due to their broad-spectrum biological activities and challenging structural complexity [13,14]. The majority of the compounds isolated to date exhibited antibiotic or antiviral activity. Tetramic acids possessing an octahydronaphthalene skeleton are rare in nature, showing activity against Gram-positive bacteria [13]. Recently, ascosetin was described as a new antibacterial tetramic acid derivative [15].

In this study, two marine strains of the family Lindgomycetaceae were shown to exhibit a diverse chemical profile, including tetramic acid derivatives having novel structures. The strains were isolated from a sponge of the Baltic Sea (Kiel Fjord) and from the Antarctic. The new compound lindgomycin (1) and ascosetin (2) showed antibiotic activities against human and plant pathogenic microorganisms.

## 2. Results and Discussion

### 2.1. Identification of Strains KF970 and LF327

**Figure 1:**
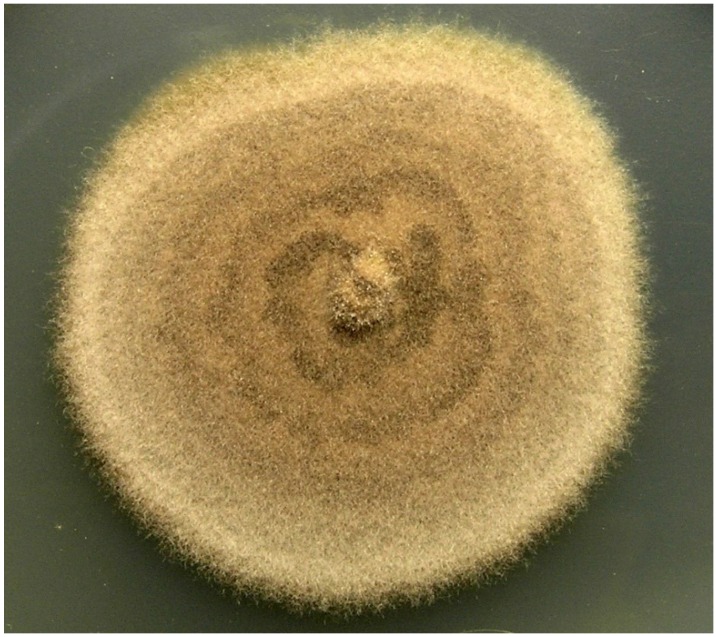
Lindgomycetaceae strain KF970, agar colony on WSP30 medium after 21 days of incubation at 22 °C.

Two fungal isolates, strains KF970 and LF327, which are members of the family Lindgomycetaceae, were isolated from different marine habitats. Both strains grew only as sterile mycelium on the media used, but did not produce conidia or ascomata on ten different media, e.g., on WSP30 (Figure 1). Thus, morphological criteria for identification of the strain were lacking. The sequences of the 18S rRNA genes comprised 1546 nucleotides for KF970 and 1065 nucleotides for LF327, respectively. Both sequences exhibited a similarity of 99.7% to each other. The highest sequence similarity (100%) was observed for the 18S RNA gene sequence to “*Phyllosticta*” *flevolandica* AFTOL-ID 1786. Nevertheless, the deposited sequence of the *P.* *flevolandica* strain comprises only approximately a fourth of the length of 18S rRNA gene (441 nucleotides) and Crous *et al*. [16] have shown that “*Phyllosticta*” *flevolandica* does not affiliate with all members of the genus *Phyllosticta*, which belong to the Botryosphaeriales.Therefore, the strains most closely related were taken into consideration for the phylogenetic classification. Among these, *Massariosphaeria typhicola* CBS 609.86 (acc. no. KF314118) showed similarities of 99.9% to KF970 and 99.5% to LF327, respectively. The 18S rDNA sequences of strains KF970 and LF327 showed 99.2%–99.5% and 99.0%–99.2% similarity to the type of strains of the seven known *Lindgomyces* species [10]. *Clohesyomyces aquaticus* MFLUCC11-0092 (acc. no. JX276949) was 99.0% similar to KF970 and 99.78% similar to LF327. A comparison with the genus *Lolia* was not possible because no 18S rRNA gene sequence was deposited.

In addition, the 28S rRNA gene fragment was analysed. The sequences had a length of 820 nucleotides (KF970) and 819 nucleotides (LF327) and showed a similarity of 99.1% to each other. The closest relative on the basis of the 28S rRNA gene sequences were *C. aquaticus* and *M. typhicola. C. aquaticus* MFLUCC11-0092 (acc. no. JX276950) exhibited a similarity of 98.9% (KF970) and 98.8% (LF327). *M. typhicola* CBS 609.86 (acc. no. EF165033) showed a similarity of 98.6% and 99.0% for KF970 and LF327, respectively. The comparison of strains KF970 and LF327 with *Lolia aquatica* (acc. no. HM367732) revealed 98.1% and 98.2% sequence similarities, respectively. Members of the genus *Lindgomyces* bore resemblance in the range of 97.3%–98.4% (KF970) and 97.4%–98.2% (LF327).

On the basis of the phylogenetic analyses, KF970 and LF327 were clearly assigned to the family Lindgomycetaceae (Pleosporales, Dothideomycetes).

### 2.2. Metabolic Profiles of the Strains KF970 and LF327

Interestingly, both Lindgomycetaceae strains, which originated from different geographic regions, produced compounds **1** and **2**. Cultivation experiments of LF327 were carried in two different media under static and non-static conditions. Mycelium and culture broth were not separated. Compounds **1** and **2** were produced in GYM4 medium. The comparison of static and non-static cultures showed that the production level in static cultures was much higher. The evaluation was done by the comparison of UV spectra and ESIMS data, obtained by analysing the extract by analytical HPLC-UV/MS.

### 2.3. Structural Elucidation

**Figure 2:**
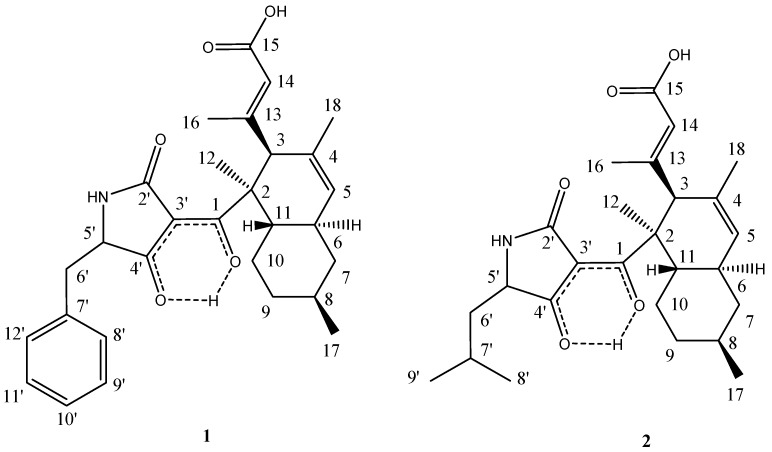
Structures of compounds **1** and **2**.

The MeOH extracts of the mycelia and the broth of strain KF970 were subjected to repeated column chromatography to purify the two polyketides **1** and **2** (Figure 2).

Compound **1** was isolated as a white powder. The molecular formula was determined to be C_29_H_35_NO_5_ by analysis of the HR-TOF-MS ion peak at *m*/*z* 478.2603 [M + H]^+^ (calcd. 478.2588). The formula was supported by the ^13^C NMR data, which showed 13 unsaturated degrees. The proton signal of H-5 appeared as a broad singlet. No observation of coupling of H-5 and H-5 indicated that the dihedral angle between H-5 and H-5 was around 90°. The NMR data (Table 1) in the upfield region of **1** were similar to those of the phomasetin and equisetin analogues [17]. These molecules contain two distinct domains, a bicyclic hydrocarbon and a tetramic acid, connected by a bridging carbonyl [13,14,15,17,18,19]. The ^13^C NMR spectrum showed the presence of 18 signals for the hydrocarbon domain including a decahydronaphthalene basic skeleton and a *sec*-butyl moiety, with the remaining 11 resonances corresponding to a heterocyclic domain including a tetramic acid basic skeleton and a benzyl moiety. When compared to the NMR data of the polyketide phomasetin, isolated from the fungus *Fusarium heterosporum*, compound **1** showed similar chemical shifts of the main decalin skeleton. However, the structures of the tetramic acid and attached unit at the decahydronaphthalene ring were less similar to phomasetin.

**Table 1:** NMR data (500 MHz) for compounds **1** and **2** in CD_3_OD.

Position	1		2	
	δ_C_^a,b^, Mult.	δ_H_^c^, Mult. (*J* in Hz)	δ_C_^a,b^, Mult.	δ_H_^c^, Mult. (*J* in Hz)
1	201.4, C		202.3, C	
2	52.2, C		52.7, C	
3α	58.0, CH	3.46, s	57.9, CH	3.52, s
4	130.8, C		130.9, C	
5	129.5, CH	5.41, br s	129.6, CH	5.43, br s
6α	39.9, CH	1.88, m	39.8, CH	1.90, m
7α	43.7, CH_2_	1.87, m	43.8, CH_2_	1.88, m
7β		0.90, m		0.86, m
8α	34.8, CH	1.54, m	34.8, CH	1.56, m
9α	37.1, CH_2_	1.73, m	37.1, CH_2_	1.67, m
9β		1.05, m		1.06, m
10α	29.6, CH_2_	0.95, m	29.6, CH_2_	1.07, m
10β		1.71, m		1.76, m
11β	41.6, CH	1.81, m	41.8, CH	1.87, m
12	15.6, CH_3_	1.38, s	15.6, CH_3_	1.49, s
13	159.8, C		159.9, C	
14	121.5, CH	5.63, s	121.6, CH	5.66, s
15	169.6, C		169.6, C	
16	17.3, CH_3_	1.91, s	17.2, CH_3_	1.97, s
17	22.9, CH_3_	0.93, d (*J* = 6.5)	22.9, CH_3_	0.92, d (*J* = 6.5)
18	22.9, CH_3_	1.54, s	22.8, CH_3_	1.55, s
2′	173.0, C		173.0, C	
3′	n.d.		n.d.	
4′	194.1, C		195.8, C	
5′	62.2, CH	4.07, dd (*J* = 6.5, 4.3)	60.4, CH	3.90, m
6′a	38.5, CH_2_	3.09 dd (*J* = 13.9, 4.3)	42.4, CH_2_	1.85, m
6′b		2.96 dd (*J* = 13.9 6.5)		0.90, m
7′	136.9, C		22.1, CH	1.50, m
8′	130.8, CH	7.19, d (*J* = 8.0)	23.9, CH_3_	0.97, d (*J* = 6.8)
9′	129.3, CH	7.24, t (*J* = 8.0)	22.89, CH_3_	0,95, d (*J* = 6.8)
10′	127.9, CH	7.21, t (*J* = 8.0)		
11′	129.3, CH	7.24, t (*J* = 8.0)		
12′	130.8, CH	7.19, d (*J* = 8.0)		

^a^ Recorded at 125 MHz; ^b^ Multiplicities inferred from DEPT and HMQC experiments; ^c^ Recorded at 500 MHz.

**Figure 3:**
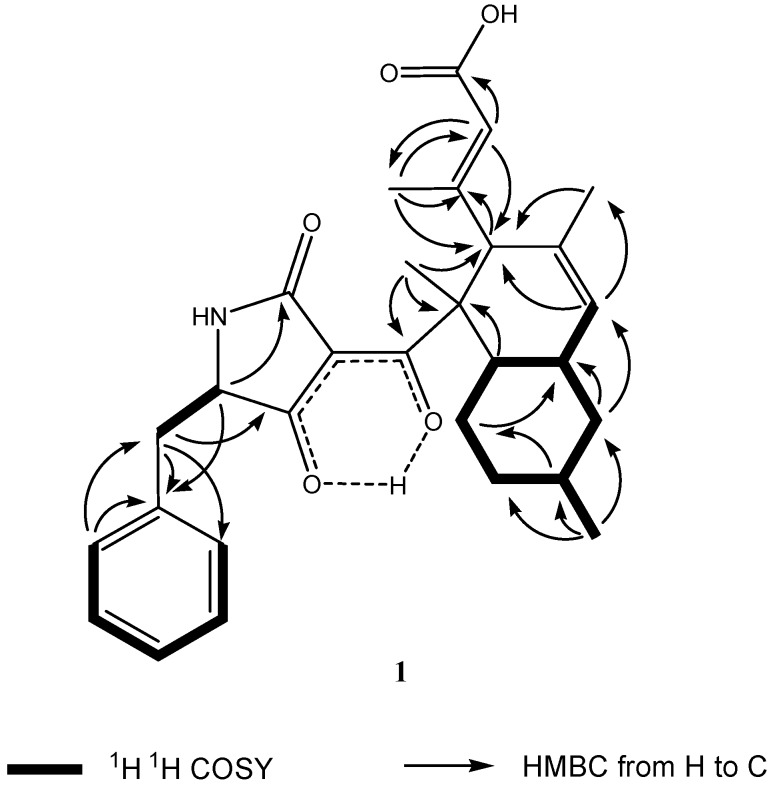
Key ^1^H–^1^H COSY and HMBC correlations of compound **1**.

The 18 signals for the hydrocarbon domain comprised four methyls (δ_C_ 15.6, 17.3, 22.9, and 22.9), three methylenes (δ_C_ 43.7, 37.1, and 29.6), four methines (δ_C_ 58.0, 39.9, 41.6, and 34.8), a quaternary carbon (δ_C_ 52.2), two sets of double bonds (130.8, 129.5, 159.8, and 121.5), a carboxylic carbon (δ_C_ 169.6), and enol carbon (δ_C_ 201.4). In the COSY spectrum of **1** (Figure 3), the methine proton at δ_H_ 1.88 (m, H-6) was coupled with another methine proton at δ_H_ 1.81 (m, H-11), the methylene protons at δ_H_ 1.87 (m, H-7α) and 0.90 (m, H-7β), and the olefin proton at 5.41, (br s, H-5). The methine proton at δ_H_ 1.54 (m, H-8) exhibited cross peaks with methylene protons of H_2_-7, H_2_-9, and methyl protons at δ_H_ 0.93 (d, *J* = 6.5 Hz, Me-17) in the COSY spectrum of **1**. The sequence of a six-member ring system of H-6/H_2_-7/H-8/H_2_-9/H_2_-10/H-11 with Me at C-8 was deduced from the above ^1^H–^1^H COSY analyses (Figure 3). HMBC analyses complete the formation of the decalin skeleton with a characteristic *sec*-butyl pedant (Figure 3). HMBC cross peaks of Me-17/C-7 and Me-17/C-9 confirmed the position of methyl group at C-8 of ring A. In the ^1^H NMR spectrum of **1**, two methyl groups displayed two singlet signals, one of which was assigned at quaternary C-2 from the observation of HMBC cross peaks of Me-12/C-1 and Me-12/C-2. The long range correlations from the proton at δ_H_ 1.54 (s, Me-18) to the double bond carbon signals at δ_C_ 130.8 (s, C-4) and δ_C_ 129.5 (d, C-5) positioned the remaining methyl at C-4. The pendant attached at C-3 was obviously different from the known phomasetin. Instead of being a penta-diene, a 3-methylbut-3-enoic acid unit was attached at C-3. The carboxylic carbon at δ_C_ 169.6 (s) was attributed to C-15 from the observation of long range correlations from the proton signals at δ_H_ 5.63 (s, H-14) to carboxylic C-15. The methyl group in the pendant was assigned at C-13 from analysis of the HMBC cross peaks from Me-16 to olefinic C-13 and C-14. The substructure of the pedant was deduced to be a 3-methylbut-3-enoic acid unit, which was positioned at C-3 from the observation of HMBC correlation from the proton at δ_H_ 3.46 (s, H-3) to the carbon at δ_C_ 159.8 (s, C-13) and HMBC correlations from the Me proton at δ_H_ 1.91 (s, Me-16) and olefinic proton at δ_H_ 5.63 (s, H-14) to the carbon signal at δ_C_ 58.0 (C-3). The two-dimensional NMR analyses mentioned above permitted the assignment of the bicyclic hydrocarbon unit of **1**.

The formula indicated that 11 resonances remained to be assigned to the tetramic acid half of molecule **1**, corresponding to a tetramic acid basic skeleton and a benzyl moiety. However, the C-3′ signal was not detected due to rapid tautomerisations to multiple enol forms [18]. In the downfield region of the NMR spectra of **1**, two sets of equivalent aromatic protons at δ_H_ 7.19, (d, *J* = 8.0 Hz, H-8′/12′) and 7.24 (t, *J* = 8.0 Hz, H-9′/11′) and an aromatic triplet at δ_H_ 7.21 (t, *J* = 8.0 Hz, H-10′) indicate that a single substituted benzene ring unit was added in the core structure of a tetramic acid moiety coupling with a decalin moiety. The structure of the five-member lactam ring at the tetramic acid unit was approved by the HMBC cross peaks of H-5′/C-2′. The five-member lactam ring proved to be linked at the quaternary C-2 via the oxygenated enolic C-1 from the observation of long range correlation from the proton signal at δ_H_ 1.38 (s, Me-12) to the enolic carbon signal at δ_C_ 201.4 (s, C-1). Owing to the formation of the intermolecular hydrogen bond between the hydroxyl at the olefin carbon and the carbonyl at the lactam ring, the C-1 was highly downshifted [14,17,19]. The benzylic methylene of H_2_-6′ showed long range two-dimensional ^1^H–^13^C correlations to the aromatic carbon C-8′/12′ and C-7′ of the phenyl ring and to the carbonyl C-4′ of the pyrrolone ring. This indicated that the benzene unit was linked with the five-member lactam unit via a methene bridge. This inference was confirmed by the observation of ^1^H–^1^H COSY cross peaks of H_2_-6′/H-5′. Thus, the planar structure of **1** was elucidated as shown in Figure 2.

**Figure 4:**
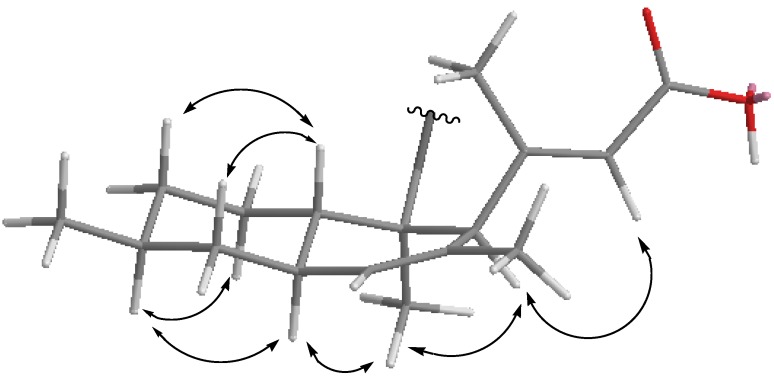
Key NOESY correlations of compound **1**.

The 1,3-diaxial NOESY cross peaks of H-6α/H-8α, H-8α/H-10α, H-11β/H-7β, and H-11β/H-9β revealed a 6,11-*trans* ring fusion between the cylcohexyl chair and cyclohexenyl boat rings of the bicyclic decalin with a β-oriented Me at C-8 (Figure 4). The NOESY correlations from Me-12 to the axial H-6a and H-3 indicated a β-oriented 3-methylbut-3-enoic acid pendant at C-3 and a β-oriented tetramic acid pendant at C-2. The diagnostic NOESY correlation from the olefinic proton at δ_H_ 5.63 (s, H-14) to the proton at δ_H_ 3.46 s (s, H-3) revealed that the stereochemistry of the double bond between C-13 and C-14 was in an *E* configuration. Thus, compound **1** is found to be an unusual polyketide and is given the trivial name lindgomycin. The combination of 5-benzylpyrrolidine-2,4-dione of compound **1** in its tetramic acid half and the 3-methylbut-3-enoic acid pendant in its decalin half allowed the assignment of a new carbon skeleton.

Compound **2** was identified as the recently described ascosetin by comparison of spectroscopic data reported by Ondeyka *et al*. [15].

### 2.4. Biological Activities

Both lindgomycin (**1**) and ascosetin (**2**) exhibited strong antibiotic activities with IC_50_ values in the range of 2 to 18 µM (Table 2). The effects against the clinically relevant bacteria *Staphylococcus epidermidis*, *Staphylococcus aureus*, methicillin-resistant *Staphylococcus aureus*, and *Propionibacterium acnes* were two times less in comparison with chloramphenicol. The activity against the human pathogenic yeast *Candida albicans* was four times lower than it was observed for nystatin. The causative agents of black rot in crucifers (e.g., cabbage) and of leaf spot disease on crops (e.g., wheat), *Xanthomonas campestris* and *Septoria tritici*, were also inhibited by the new compounds. No inhibition of Gram-negative bacteria was observed.

**Table 2:** Antibiotic profiles of compounds **1** and **2**. The IC_50_ values are given in µM.

Test Strain	1	2	Positive Controls
*B. subtilis*	2.2 (±0.6)	3.4 (±1.1)	chloramphenicol: 1.45 (±0.13)
*X. campestris*	17.8 (±1.6)	14.8 (±0.7)	chloramphenicol: 2.88 (±0.9)
*S. epidermidis*	4.6 (±0.8)	6.3 (±0.7)	chloramphenicol: 1.81 (±0.04)
*S. aureus*	2.7 (±0.56)	2.9 (±1.1)	chloramphenicol: 1.59 (±0.07)
*S. aureus* (MRSA)	5.1 (±0.2)	3.2 (±0.4)	chloramphenicol: 2.46 (±0.04)
*C. albicans*	5.7 (±0.9)	8.0 (±1.4)	nystatin: 1.71 (±0.28)
*S. tritici*	5.1 (±0.7)	10.0 (±3.1)	nystatin: 0.76 (±0.23)
*P. acnes*	4.7 (±0.4)	2.8 (±0.7)	chloramphenicol: 1.01 (±0.01)
*E. coli*	>100	>100	chloramphenicol: 373 (±0.10)
*P. aeruginosa*	>200	>200	chloramphenicol: 8.86 (±0.36)

### 2.5. Biotechnological Scale Up

In order to insure sustainable production of **1** and **2** in larger amounts, we have performed experiments to optimise and scale up the biotechnological production from Erlenmeyer flask cultures to controllable stirred tank reactors. As other fungi, strain KF970 showed variation in the metabolite production in various media and growth conditions. Among a number of culture media, the best production of **1** and **2** was found in GYM4 medium and in standing cultures as well as in shaken Erlenmeyer flasks at pH 7.2 and in the absence of added salt to the medium.

Interestingly, the culture medium and the age of the pre-cultures were highly important for good growth and production. GYM4 was the best medium for the pre-cultures, but if these were older than 21 days, no significant production could be obtained.

Based on the content of nitrogen in the candidate structures, a medium containing a high nitrogen/carbon ratio was introduced in order to further improve the production rate. Casamino acids medium proved to support very good growth in small brown pellets and improvement of the production (compared to GYM4) of 2–4 times. This was observed in standing cultures but more pronounced in shaken Erlenmeyer flasks.

For a sustainable large scale production, the transfer of the production into a fermenter system (stirred tank reactor) is essential. Therefore, the best conditions for the production of **1** and **2** by strain KF970 in Erlenmeyer flasks were used to establish the production in a 10 L fermenter. Low pH turned out to be best for production of secondary metabolites by strain KF970, and for all subsequent experiments in STR, the pH value was set to pH 5. The fungus showed comparable growth behavior in a 10 L fermenter system with controlled pH and air saturation (minimum value set to 30%), reaching the exponential phase of growth after 3 days of cultivation. Both the low pH and stable oxygen levels in the medium due to the stirring stimulate hyphal growth. Production of **1** and **2** occurred within the exponential growth phase with a maximum after 7 days of cultivation. This is a significant reduction of cultivation time for production as compared to a minimum of 14 days needed to reach maximum product levels of both compounds in Erlenmeyer flasks. After this period, growth of the fungus continued, but concentrations of **1** and **2** slowly decreased (with a half-life of 6 days). Considering the optimal time point for harvest, the production yield of **1** and **2** increased from 0.2 mg/L in Erlenmeyer flasks to 5.0 mg/L in a 10 L fermenter.

## 3. Experimental Section

### 3.1. General Experimental Procedures

Optical rotations were recorded on a Perkin Elmer 241 polarimeter. The IR spectra were run on a Perkin Elmer spectrometer with an attenuated total reflectance (ATR) unit. ^1^H NMR (500 MHz) and ^13^C NMR (125 MHz) spectra were measured at 25 °C on a Bruker AVANCE DMX 500 NMR spectrometer with tetramethylsilane (TMS) as internal standard. The signals of the residual solvent protons and the solvent carbons were used as internal references (δ_H_ 3.31 ppm and δ_C_ 49.0 ppm for methanol-*d*_4_). High-resolution mass spectra were acquired on a benchtop time-of-flight spectrometer (micrOTOF II, Bruker Daltonics, Germany) with positive electrospray ionization (ESI).

### 3.2. Isolation, Cultivation, and Storage of the Producer Strains KF970 and LF327

The fungus KF970 was isolated from samples of the expedition of RV Polarstern to the Arctic in 1991 by K. Schaumann [20]. The strain LF327 was isolated from the sponge *Halichondria panicea,* which was collected at the Kiel Fjord (Baltic Sea, Germany). Both strains were grown on WSP30 agar, a modified Wickerham-medium consisting of 1% glucose, 0.5% peptone, 0.3% yeast extract, 0.3% malt extract, 3% sodium chloride (pH = 6.8) [21]. The strains were stored in liquid nitrogen and in the Microbank System at −80 °C (MAST DIAGNOSTIKA, Reinfeld, Germany).

### 3.3. Identification of the Strains KF970 and LF327

In order to obtain morphological characteristics for the identification of the isolate KF970, it was cultivated on the following 10 agar media to stimulate the formation of conidia or ascomata. GPY medium: 0.1% glucose × H_2_O, 0.05% peptone, 0.01% yeast extract, 1.5% agar, North Sea water, pH 7.3 ± 0.1; WM medium: 1% glucose × H_2_O, 0.5% peptone, 0.3% yeast extract, 0.3% malt extract, 3% sodium chloride, 1.5% agar, pH 6.8; Czapek-Dox medium (Merck, Darmstadt, Germany): 3% sucrose, 0.3% sodium nitrate, 0.05 magnesium sulfate, 0.05 potassium chloride, 0.001% iron(II)sulfate, 0.1% di-potassium hydrogen phosphate, 1.5% agar, pH 7.3; ME medium: 1.7% malt extract, 1.5% sodium chloride, 1.5% agar, pH 4.5; SM medium: 2.0% soja peptone, 1.5% sodium chloride, 2.0% mannit, 1.5% agar, pH 6.9; PDA medium: 0.4% potato extract, 2.0% glucose × H_2_O, 1.5% agar, pH 4.7; MB medium: 3.74% Bacto Marine Broth (Difco 2216, Becton Dickinson and Company, Heidelberg, Germany), 1.5% agar, pH 7.6 ± 0.2; TSB3 + 1% NaCl: 0.3% BD BBL™ Trypticase™ Soy Broth (Becton Dickinson and Company, Heidelberg, Germany), 1% NaCl, 1.5% agar, pH 7.2; Carrot medium: 100 g grinded carrot, 1.5% agar, pH 6.5. If not otherwise indicated the media were adjusted with aqua dest. to 1 L.

For the genetic characterisation of the fungus the 18S rRNA and 28S rRNA gene sequences were analysed. DNA-extraction was performed using the Precellys 24 system (Bertin Technologies, Paris, France) according to [22]. Specific PCR for amplification of fungal 18S rRNA and 28S rRNA gene fragments was carried out using puReTaq™ Ready-To-Go™ PCR Beads (GE Healthcare, Munich, Germany). The PCR of the 18S rDNA sequence was performed using the primers NS1 (5′-GTAGTCATATGCTTGTCT-3′) and FR1 (5′-AICCATTCAATCGGTAIT-3′) according to Gomes *et al*. [23]. PCR was conducted as follows: initial denaturation (8 min at 94 °C), 35 cycles of primer denaturation (30 s at 94 °C), annealing (45 s at 48 °C), and elongation (3 min at 72 °C), followed by a final elongation step (10 min at 72 °C). Since most of the phylogenetic studies which, based on the large-subunit RNA, have been performed with the first 800–900 nucleotides, the primers used for the amplification of the 28S rDNA fragment (5.8S region, ITS2 and part of the large-subunit RNA) were 5.8SR (5′-TCGATGAAGAACGCAGCG-3′) and LR7 (5′-TACTACCACCAAGATCT-3′) [24]. The conditions for the PCR were: initial denaturation (3 min at 94 °C), 35 cycles of primer denaturation (60 s at 94 °C), annealing (30 s at 55 °C), and elongation (2 min at 72 °C) followed by a final elongation step (5 min at 72 °C) [25]. The PCR products were checked for correct length, 1650 nucleotides and 1414 nucleotides, respectively, using a 1% agarose gel in 1× TBE buffer (8.9 mM Tris, 8.9 mM borate, 0.2 mM EDTA) and the DNA molecular weight marker X (Roche, 0.07–12.2 kbp). PCR products were sequenced using the ABI PRISM^®^ BigDye™ Terminator Ready Reaction Kit (Applied Biosystems) on an ABI PRISM^®^ 310 Genetic Analyzer (Perkin Elmer Applied Biosystems, Waltham, Massachusetts, USA). The sequence of the almost-complete 18S rRNA gene was determined with the primers NS1, 470F (5′-CAGCAGGCGCGCAAATTA-3′; Dr. Sven Neulinger, pers. Mitteilung), and FR1. The LR0R (5′-ACCCGCTGAACTTAAGC-3′) and LR5 (5′-TCCTGAGGGAAACTTCG-3′) primer were used for sequencing the entire 28S rRNA gene fragment [25]. Closest relatives were identified by sequence comparison with the NCBI Genbank database using BLAST (Basic Local Alignment Search Tool) [26]. Sequence similarity values were determined with the “bl2seq” tool of the NCBI database [27].

### 3.4. Comparison of the Metabolic Profiles the Strains KF970 and LF327

Cultivation of both strains was carried out in two L Erlenmeyer flasks containing 750 mL GYM4 (0.4% malt extract, 0.4% yeast extract, 0.4% glucose × H_2_O, 0.2% CaCO_3_, pH 7.2) for 21 days at 28 °C as static and shaken cultures in the dark. Circular agar pieces (WSP30, diameter of 1.8 cm) were used for inoculation. Extraction of mycelium and culture broth was performed using ethyl acetate added to the cultures in equal volume. The metabolite profiles of both strains were evaluated for compounds **1** and **2** using analytical HPLC-UV/MS.

### 3.5. Fermentation and Production of Extracts for the Purification of Compounds **1** and **2**

Strain KF970 was inoculated onto agar-plates containing WSP30 medium. After incubation for 15 days at 22 °C the pre-culture was used for inoculation of two L Erlenmeyer flasks containing 750 mL SM medium (2% soy peptone, 2% mannitol, 1.5% NaCl, pH 7.0). The flasks were incubated for 32 days at 28 °C as static cultures in the dark. The mycelium was separated from the culture medium. The 22 L fermentation broth was extracted using ethyl acetate (12 L). After evaporation of the solvents the crude extract was re-dissolved in 5 mL methanol and stored at 4 °C until further use.

### 3.6. Scale up Production

For media optimisation, cultures were grown in 100 mL medium (in 300 mL EMK) and inoculated with mycelium pieces (2, ø~1 cm). The following media were used: SA (Sabouraud-Agar, 2% glucose, 1%, pH 5.6), GYM4 (0.4% glucose, 0.4% yeast extract, 0.4% malt extract, 0.2% CaCO_3_, pH 7.2), Czapek Dox (CD, 3% sucrose, 0.3% sodium nitrate, 0.1% K_2_HPO_4_, 0.05% MgSO_4_, 0.05% KCl, 0.001% iron(II)sulfate, pH 7.0), WSP30 (1% glucose, 0.5% soy peptone, 0.3% malt extract, 0.3% yeast extract, pH 6.6 +/− Tropic marine 3%), ME (1.7% malt extract, 1.5% NaCl), PDA (Potato-Dextrose-Agar BD (3.9%) or Bouillon (2.65%), pH 5.6), and SM (2% soy peptone, 2% mannitol, 1.5% NaCl, pH 6.7). After incubation at 28 °C for 14–24 days, growth and production status were estimated. Production was measured using the HPLC-DAD-MS method described above after extraction with a minimum of 100 mL ethyl acetate. For optimisation of cultivation time, preculture medium, and introduction of casamino acids, the following setups were chosen. (A) cultivation time GYM4 (standing and shaken cultures): 6 × 750 mL GYM4 (in a 2 L EMK) were inoculated with 6–8 mycelium pieces (ø ~1 cm, 14 days, WSP30), incubation: 28 °C, stand + 120 rpm, 14 days, 21days , 28 days; (B) variation of preculture medium: 6 × 750 mL GYM4 (2 L EMK) were inoculated with 6–8 mycelium pieces (ø ~1 cm, 14 days, GYM4 or PDA), incubation: 28 °C, stand + 120 rpm, 14 days, 21 days, 28 days; (C) casamino acids as medium: 2 × 750 mL casamino acids (in 2 L EMK) were inoculated with 6–8 mycelium pieces (ø~1 cm, 14 days, WSP30), incubation: 28 °C, stand + 120 rpm, 14 days, 21 days, 28 days. All cultures were extracted after separation of culture medium and mycelium by addition of ethanol (cells) or ethyl acetate (culture supernatant). For subsequent optimisation, GYM4 and casamino acid medium (0.25% caseinhydrolysate, 4% glucose, 0.01% MgSO_4_·7H_2_O, 0.18% KH_2_PO_4_, pH 6.8) were used for incubation at 28 °C, 120 rpm or stand, for 15 days. In the 10 L STR system (Biostat, Braun, Melsungen, Germany), pH, oxygen, CO_2_-outlet, and stirring speed were controlled. The oxygen content in the medium was set to a minimum of 30% air saturation. Foam formation was stopped by addition of antifoam (Sigma, Taufkirchen, Germany). After cultivation, cells were separated from the culture broth by means of centrifugation. For the 1 L and 10 L scale, culture supernatant and cells were extracted by addition of two volumes ethyl acetate. The organic solvent was separated and concentrated to dryness under reduced pressure.

### 3.7. Extraction and Isolation of Compounds **1** and **2**

Analytical reversed phase HPLC-DAD(UV)-MS experiments were performed using a C_18_ column (Phenomenex Onyx Monolithic C18, 100 mm × 3.00 mm) applying an H_2_O/acetonitrile (ACN) gradient with 0.1% formic acid added to both solvents (gradient: 0 min 5% ACN, 4 min 60% ACN, 6 min 100% ACN; flow 2 mL/min each; 6.1 min 100% ACN, 6.8 min 100% ACN, 7.0 min 5% ACN, 8.2 min 5%; flow 2.5 mL/min each) on a VWR Hitachi Elite LaChrom system with an L-2450 diode array detector, an L-2130 pump, and an L-2200 autosampler (VWR, Darmstadt, Germany). The system was coupled to an ESI-ion trap detector with positive ionization (Esquire 4000, Bruker Daltonics, Germany) for mass detection. Compounds **1** and **2** eluted with a retention time of 5.9 min and 6.0 min, respectively.

The preparative HPLC was conducted with a HPLC-UV system (VWR International LaPrep, pump P110, UV detector P311, smartline 3900 autosampler) coupled with a LABOCOL Vario-2000 fraction collector (LABOMATIC, Weil am Rhein, Germany) and a C_18_ column (Phenomenex Gemini-NX C18 110A, 100 mm × 50 mm). The gradient parameters were: 0 min 10% acetonitrile (ACN), 17.5 min 60% ACN, 22 min 100% ACN, 25 min 100% ACN, 26 min 10% ACN, 28 min 10% ACN; flow: 100 mL/min).

Semi-preparative HPLC was carried out using a HPLC-UV system (VWR Hitachi Elite LaChrom system, L-1230 pump, L-2450 diode array detector, L-2200 autosampler, Phenomenex Gemini-NX C18 110A, 100 mm × 50 mm, column). For the preparation of compound **1** and **2**, ACN was used, with a gradient from 65% ACN increasing to 85% in 20 min and 70% ACN increasing to 90% in 20 min, respectively, and a flow of 15 mL/min. The compounds eluted with a retention time of 9.1 min and 8.4 min, respectively. The yield was 8.0 mg of compound **1** and 3.7 mg of compound **2**.

Lindgomycin (1): white powder; ${\left[\alpha \right]}_{\text{D}}^{20}$ −42 (*c* 0.1, CHCl_3_); UV (MeOH) λ_max_ (log ε) 254 (4.17), 293 (4.50) nm; IR ν_max_ 2913, 1630, 1556, 1453, 1378, 1150, 1026, 878, 790, 699, 611 cm^−1^; ^1^H NMR and ^13^C NMR, see Table 1; ESIMS *m*/*z* 478 [M + H]^+^; HR-TOF-MS *m*/z 478.2603 [M + H]^+^ (calcd. for C_29_H_36_NO_5_, 478.2588).

### 3.8. Antibiotic Activities Assays

The antimicrobial activities of compounds **1** and **2** against the bacteria *Bacillus subtilis* (DSM 347), the plant pathogen *Xanthomonas campestris* (DSM 2405), as well as the human pathogenic yeast *Candida albicans* (DSM 1386) were determined according to Ohlendorf *et al*. [28]. *Escherichia coli* K12 (DSM 498) and *Pseudomonas aeruginosa* (DSM 50071) were used in the same manner as *B. subtilis.* The bioassays with the clinically relevant bacterial strains *Staphylococcus epidermidis* (DSM 20044), methicillin-resistant *Staphylococcus aureus* (MRSA) (DSM 18827), and the causative agent of acne, *Propionibacterium acnes* (DSM 1897^T^), were performed as described by Silber *et al*. [29]. *Staphylococcus aureus* (DSM 346) was investigated in the same manner as *S. epidermidis*. The phytopathogenic fungus *Septoria tritici* was tested according to Jansen *et al.* [30].

## 4. Conclusions

Only little information is available on the production of secondary metabolites by members of the fungal order Pleosporales. The isolate *Nodulisporium* sp. CRIF2 from an unidentified soft coral (Surin Island, Thailand) produced the new compounds (*Z*)-6-benzylidene-3-hydroxy-methyl-1,4-dimethyl-3-methylsulfanylpiperazine-2,5-dione, which exhibited weak cytotoxic activity, and (3*S*,3′*R*)-3-(3′-hydroxybutyl)-7-methoxyphtalide, as well as the known substances (*S*)-3-butyl-7-methoxyphtalide, (3*R*,6*R*)-bisdethiodi(methylthio)-hyalodendrin, and bis-*N*-norgliovictin [31]. Nodulisporacid A, and derivatives thereof, and the tetramic acid vermelhotin were shown to be produced by the Pleosporales strain CRI247-01, which was derived from an unidentified sponge [32]. Nodulisporacid A and vermelhotin inhibited *Plasmodium falciparum,* the causative agents of malaria tropica.

Xanthoquinodine A3 and B3 were produced by *Humicola* sp. and showed anticoccidial (A3 and B3) and antibacterial (A3) activity [33]. Antimycobacterial properties were exhibited by chaetomanone, a product from *Chaetomium globosum* [34]. In addition, some metabolites with unknown bioactivities were described: Paecilin A from a mangrove derived *Paecilomyces* sp. strain, the pigment ergoxanthin from *Claviceps purpurea,* and the ergochromes blennolide D and G from *Blennoria* sp. [35,36,37].

Polyketides **1** and **2** were shown to contain two distinct domains, a bicyclic hydrocarbon and a tetramic acid, connected by a bridging carbonyl. The tetramic acid substructure of compound **1** was proved to possess a unique 5-benzylpyrrolidine-2,4-dione unit. Both compounds, lindgomycin (**1**) and ascosetin (**2**), showed promising antibiotic inhibitory activities against clinically relevant microorganisms, in particular Gram-positive bacteria, yeasts, and fungi, such as methicillin-resistant *S. aureus* (MRSA), *P. acnes*, *S. tritici*, and *C. albicans.* The lack of activity against Gram-negative bacteria has already been noticed for other tetramic acid derivatives by Lowery *et al*. [38] and maybe related to the difference in the cell wall structures. Apparently the outer membrane of Gram-negative bacteria is an efficient permeability barrier for these molecules.

The WHO stated that 25,000 persons die each year due to infections with antibiotic resistant bacteria in 29 European countries [39]. Specifically, MRSA strains are causing infections with high mortality rates and a growing rate of resistance [40]. According to the data raised by the European Antimicrobial Resistance Surveillance Network (EARS-Net), the percentage of MRSA-positive isolates exceed 20% in 11 of 28 European countries that participate at the EARS-Net [41]. In addition to the highly demanded search for new natural products active against antibiotic-resistant bacteria, a number of other health issues also require new active compounds for improved treatment. These include new molecules against the human pathogens *P. acnes* or *C. albicans*. *P. acnes* is involved in the inflammatory skin disease acne vulgaris and causes opportunistic human infections [42]. *Candida* species are responsible for the infectious disease candidiasis, especially in immuno-compromised patients [43]. More than 90% of invasive infections are caused by *C. albicans*. Without a doubt, there still remains a strong demand for new antibiotics that might be more effective, less toxic, and show a lower risk of resistance. Lindgomycin (**1**) as well as ascosetin (**2**) could be promising candidates in this search.
